# Major depressive disorder seven years after the conflict in northern Uganda: burden, risk factors and impact on outcomes (The Wayo-Nero Study)

**DOI:** 10.1186/s12888-015-0423-z

**Published:** 2015-03-14

**Authors:** James Mugisha, Herbert Muyinda, Samuel Malamba, Eugene Kinyanda

**Affiliations:** Makerere University, Child Health and Development Center, School of Health Sciences, P. Box 6717, Makerere Hill, Kampala, Uganda; Butabika National Psychiatric Referral Hospital, P.o.Box 7017, Off Old Port Bell, Kampala, Uganda; Sør-Trøndelag University College, E. C. Dahls gate 2, 7012 Trondheim, Norway; Uganda Virus Research Institute (UVRI) HIV Reference Laboratory Program, 50-59 Nakiwogo Street, Entebbe, Uganda; MRC/UVRI Uganda Research Unit on AIDS, Uganda/MRC-DFID African Leadership Award, 50-59 Nakiwogo Street, Entebbe, Uganda; Department of Psychiatry, Makerere University College of Health Sciences, School of Health Sciences, Makerere Hill, Kampala, Uganda; London School of Hygiene & Tropical Medicine, Keppel Street, London, WC1E, 7HT UK

**Keywords:** Major depressive disorder, Post-conflict, Northern Uganda, Risk factors, Outcomes

## Abstract

**Background:**

Major depressive disorder (MDD) is a major public health burden in conflict areas. However, it is not known for how long and by how much the observed high rates of MDD seen in conflict settings persist into the post-conflict period.

**Methods:**

A cross sectional survey was employed seven years after the conflict in northern Uganda had ended in the three districts of Amuru, Gulu and Nwoya.

**Results:**

The prevalence of major depressive disorder (MDD) was 24.7% (95% CI: 22.9%-26.4%). The distribution by gender was females 29.2% (95% CI: 14.6%-19.5%) and males 17.0% (95% CI: 26.9%-31.5%). The risk factors for MDD fell under the broad domains of socio-demographic factors (female gender, increasing age, being widowed and being separated/divorced); distal psychosocial vulnerability factors ( being HIV positive, low social support, increasing war trauma events previously experienced, war trauma stress scores previously experienced, past psychiatric history, family history of mental illness, negative coping style, increasing childhood trauma scores, life-time attempted suicide, PTSD, generalized anxiety disorder and alcohol dependency disorder) and the psychosocial stressors (food insufficiency, increasing negative life event scores, increasing stress scores). ‘Not receiving anti-retroviral therapy’ for those who were HIV positive was the only negative clinical and behavioral outcome associated with MDD.

**Conclusions:**

These findings indicate that post-conflict northern Uganda still has high rates for MDD. The risk factors are quite many (including psychiatric, psychological and social factors) hence the need for effective multi-sectoral programs to address the high rates of MDD in the region. These programs should be long term in order to address the long term effects of war. Longitudinal studies are recommended to continuously assess the trends of MDD in the region and remedial action taken.

## Background

Globally, non-communicable diseases such as mental illness, neurological and substance abuse disorders are recognized as a major public health burden amidst weak public health systems in post-conflict areas [1]. It has been observed that exposure to chronic civil conflict that is characterized by widespread human suffering and massive displacement is associated with high rates of major depressive disorder (MDD) of between 39% and 97% [2-8]. It is however not known for how long and by how much these rates of MDD persists in the post-conflict situation- when there is cessation of conflict and the communities have gone back to a semblance of ordinary life. Bolton and colleagues [9] reported a rate of MDD of 15.5% five years after the genocide in Rwanda while Wong and colleagues [10] among Cambodian refugees assessed two decades after resettlement in the USA reported a rate of MDD 51%. In the latter case the situation of war trauma may have been confounded by acculturative stress which has been associated with depression [11].

Several factors have been reported to be associated with MDD in conflict settings and these include: female gender [2-4,8,12-14]; indices of socio-economic disadvantage-widowhood, disability, being married for women, unemployment, no formal education, abject poverty, broken families, long periods of displacement, experiencing ill health without medical care [2,3,8,13]; and degree of exposure to traumatic events [2-5,8,13,15]. Apart from exposure to traumatic events reported by Wong and Colleagues [10], it is not known which other factors continue to contribute to the risk of MDD in the post-conflict situation after war has ceased and communities are re-settled.

MDD in conflict settings has been associated with impaired functioning [7,9] but it is not known for how long this persists in the post-conflict situation. Yet, this information is important in planning effective post conflict recovery programs, including public health interventions. It is also vital that the planning process for interventions in post areas is based on accurate data to improve program effectiveness.

In this study we undertook a community survey in post-conflict northern Uganda seven years after the 20 year civil conflict between the Lord’s Resistance Army and the central government army (Uganda Peoples Defense Forces; UPDF) had ended, the population had gone back to their villages and the government is implementing a post-conflict re-construction program for the region. This conflict had led to tremendous suffering of the population including the forced displacement of over 2 million people (80% of the population in the region) into squalid living conditions in internally displaced persons camps; abduction of more than 20,000 children; and mass sexual traumatization of the population [16-21]. Studies of psychiatric disorder undertaken in this region during the conflict reported rates of MDD depression of between 31% and 67% with no known studies to date undertaken since the conflict ended [5,8]. Additionally, previous studies examined a limited range of risk factors for MDD, used non diagnostic tools to make psychiatric diagnoses and never examined the association between MDD and important negative clinical and behavior outcomes.

To address some of these methodological issues, we assessed for psychiatric disorder using a DSM IV based diagnostic tool, the Mini-International Neuropsychiatric Interview- M.I.N.I. [22] administered by trained psychiatric nurses. In this study we explored for the association between psychiatric disorder (including MDD) and a number of socio-demographic, social, clinical and psychological constructs. Our interest in exploring this wide range of risk factors is based on the notion that several studies conducted in the region after the war indicate that the population continues to be exposed to a wide range of psychosocial vulnerability factors [13,23]. We also explored the relationship between psychiatric disorder with key psychiatric, clinical and behavior outcomes. In this paper we present our findings on major depressive disorder.

## Methods

### Study design and setting

This community survey was nested into a bigger project that is delivering a novel community based intervention that is using kinship relationships (Wayos [aunties] and Neros [uncles]) to increase the uptake of mental health services in post-conflict northern Uganda (Wayo-Nero) [24]. In this study we employed a cross sectional survey design where a representative sample was drawn from three mid- northern Uganda districts of Gulu, Amuru and Nwoya. These three districts were some of the most affected by the 20 year civil war in northern Uganda. Through a consultative process with community leaders at the district level of each of the selected study district, all the sub-counties in a given district were rated on how badly (intensity) affected they were by the civil conflict from the most affected to the least affected (the purpose of this grading process was to select the sub-counties which were to be involved in the main interventional study, and the same sub-counties were used in this survey). In each district, two sub counties rated as most affected by the civil war and having a health center III (a health centre at the level of the sub-county) or IV (a health centre at the level of a country) in their midst (a requirement for the main Wayo-Nero intervention) were selected. The sub-counties selected for this study were Lalogi and Koro (in Gulu district); Amuru and Atiak (in Amuru district); and Kochgoma and Alero (in Nwoya district).

### Sampling

A multistage sampling method was used to select study respondents. Study respondents had to be residents in the selected sub-counties and 18 years and above in age. In each of the selected sub-counties a parish was randomly selected for inclusion in this study. A list of villages in each of the selected parishes was developed. All villages in the selected parishes were included in this study. Since there was no reliable source of information on the number of households in each village and on the number of adult individuals in each household, we could not develop a sampling frame based on households or individuals. Using the health centre III or IV as the central point in each parish, teams of research assistants fanned out in the 4 directions of the compass to interview study respondents. All adult members (18 years and above) in homesteads lying along a village road in one direction of the compass were visited and interviewed for this study by one of the teams of research assistants. All households within a particular direction were exhausted first before taking another direction. During this movement, both near and far households from the road side were selected. However, we excluded from interviews all community members who had not stayed in the area for more than six months and, those who could not fluently speak Luo (the local dialect and could not respondent to the translated tools). We also excluded those from the survey, community members that were drunk or too sick. In each household, we interview all the adult members who met the inclusion criteria. In case someone was missing at the time of the interviews, call back visits were made. However, due to resource limitations we could not make more than two call back visits to the same household. We did not find any refusals during data collection possibly because we were working alongside community gatekeepers-community leaders. Interviews in each parish were conducted until a total number of 400 study respondents had been attained (in 2 sub-counties the selected parish could not provide all the required 400 respondents so data collection was extended into the neighboring parish of the same sub-county). Study interviewers were all psychiatric nurses who had been trained on the objectives of the study, consenting procedures, administration of the study questionnaire and the referral process for those respondents found to have significant psychiatric problems.

### Sample size calculation

A multistage sampling procedure was used to draw up a representative sample from each of the six study sub-counties. A sample size of 400 respondents per sub-county was calculated using the formula: $n={\left(\frac{Z}{m}\right)}^2\widehat{p}\left(1-\widehat{p}\right)\times \frac{deff}{R}$ (Kirkwood and Sterne, [25]). To take care of possible non-response (R) and sampling design (*deff*), assuming a design effect (*deff*) of 2 and a non-response rate (R) of 0.987%, ‘*m*’ level of precision desired. A sample size (n) of 2,400 was calculated to produce a two-sided 95% confidence interval ranging from 26.7% to 31.9% when the sample percentage (P) is 29.3%.

### Data collection tools

A structured questionnaire consisting of various standardized tools previously employed in the Ugandan context by one of the investigators (EK) was used in this study [19,26,27]. The different assessment tools in the study questionnaire we translated into the local Luo language by a process of forward and backward translation by two teams of mental health professionals working independently of each other. A consensus meeting was then held to resolve those items where there was a wide disparity in the two translations.

*The sections of the study questionnaire were:*

1. Ecological factor*: The sub-county*
2. Socio-demographic factors: *sex, age, marital status, highest educational attainment, religion, and employment status.*
3. Social factors i). *‘Food insecurity’* (assessed by the question, ’in the last month, did you or your family have enough food?’); ii) *‘Negative life events index’*, constructed from items of the adverse life events module of the European Parasuicide Interview Schedule [28] that has previously been modified for the Ugandan situation by Kinyanda and colleagues [26]. A total score was generated to reflect the total number of life events reported; iii) ‘*War trauma events index’*: These items were derived from the commonly reported forms of war trauma in Uganda including loss of loved ones events, sexual trauma events, physical trauma events and psychological trauma events [19,29]; iv) *‘Stress Score indices’*, were constructed both for ‘negative life events’ and for ‘war trauma events’. Each of the items of the modules on ‘negative life events’ and for ‘war trauma experiences’ was scored on a 3 point Likert scale where respondents were asked the question, ’how stressful did you find the event?’ with possible responses being: 0 = (not stressing/minimal stressing), 1 = (moderately stressing), 2 = (severely stressing). A total score was generated for each module where high scores reflected more stress; v) *Social support index*’ was assessed using the Multidimensional Scale of Perceived Social Support (MSPSS) [30]. The MSPSS is a 12-item instrument that was designed to assess perceptions about support from family, friends and a significant other. Originally designed to have each item on this scale graded according to a 7 point Likert Scale, this was shortened to 5 choices namely, 1 = ‘strongly disagree’; 2 = ‘mildly disagree’; 3 = ‘mildly agree’; 4 = ‘strongly agree’; 5 = ‘if you very strongly agree’ as some choices did not make sense in the local cultural context. The α-Cronbach of this scale in this study was 0.92.
4. Psychological and clinical factors: i) *Psychiatric disorders* were assessed using the M.I.N.I. neuropsychiatric interview (MINI Plus) which is a modular DSM IV based structured interview [22]. The psychiatric disorder modules used in this study were: major depressive disorder, suicidality, alcohol dependency/ abuse disorders, generalized anxiety disorder and post-traumatic stress disorder; ii) *Family history of psychiatric disorder* was assessed by the question, ‘has any member of your family (immediate family- parent, sibling, child) ever been diagnosed with a psychiatric disorder?’; iii) *‘Past psychiatric history’* by asking the question, ‘have you ever suffered from any nervous or psychiatric condition?’; iv) *Adverse life events in childhood* was assessed using the Childhood Trauma Questionnaire-Short Form (CTQ-SF) [31]. This is a 28 item questionnaire that asks about negative life events experienced as a child and as an adolescent. The respondent is required to respond based on a 5 point Likert format where 1 = ‘never true’; 2 = ‘rarely true’; 3 = ‘sometimes true’; 4 = ‘often true’; 5 = ‘very often true’. A total score was generated. To ensure that all the items of the CTQ-SF are scored in the same direction, the following items were reverse scored [2,5,7,10,13,16,19,22,26], and 28. The α-Cronbach of this scale in this study was 0.89; v) *Positive Coping style index*. This was assessed using the Mental Adjustment to Cancer Scale (MAC) [32] whose items have been previously adapted to the Uganda situation [33]. Each of this scale's 17 questionnaire items is scored on a 4-item Likert scale 1 = (definitely does not apply to me), 2 = (does not apply to me), 3 = (applies to me), 4 = (definitely applies to me). To score all the questionnaire items so that they are all in the same direction i.e. higher scores reflecting less negative coping style, questionnaires items that were cast negatively were reverse scored at analysis. A total score was generated so that higher scores reflected a more negative coping style, the α-Cronbach of this scale in this study was 0.93; vi) *Self report on HIV status*. This was assessed by asking the question, ‘what is your HIV status’.
5. Clinical and behavioral outcomes. The following clinical and behavioral outcomes were assessed in this study: i)*‘Whether currently receiving treatment for psychiatric illness’* by asking those who reported a past history of ever suffering from any nervous or psychiatric condition the question, ‘are you currently receiving treatment for the above condition’; ii) for those who are HIV positive, *‘whether they were receiving HIV treatment’* by asking the question, ‘are you receiving treatment for HIV/AIDS ?’; iii) A *High risk sexual behavior index* was constructed by asking respondents whether in the past month they had engaged in high risk sexual practices that have been associated with HIV transmission in the Ugandan cultural context as reported by various HIV sero-behavioral surveys [33-35], the sexual behavioral practices assessed were: ‘have you had sex with anyone other than your regular partner?’, ‘have you engaged in sex in exchange for gifts/money?’, ‘have you engaged in forced sex including rape?’, ‘have you engaged in sex with someone much older than you who is not your partner?’, ‘have you engaged in sex with someone you have just met?’. The high risk sexual behavior index was positive if a respondent reported at least one of these items.

### Statistical analysis

Data was analyzed using STATA software (StataCorp LP, 4905 Lakeway Drive, College Station, TX, USA). Logistic regression models were used to assess univariate associations between the dependent variable (major depressive disorder) and the independent variables including socio-demographic, social, psychological and clinical factors. Unadjusted Odds Ratio and those adjusted for sex and age are reported. Analyses accounted for the multi-sampling design of the study including stratification, unequal selection probabilities, and clustering. Survey analyses using the ‘svyset’ command and the ‘svy’ prefix/procedures in STATA were used to account for the study design. The primary sampling unit (PSU) was the parish and the stratification variables include the 9 strata (6 certainty primary frame sub-county unit strata plus three district strata). The sampling weights were based on the selection probabilities at each level of selection. All factors associated with MDD at a level of significance of ≤0.1 in the bivariate models were entered into a multivariable model to determine their independent effect on MDD. A backward elimination method that dropped, at each step, all variables that did not reach the <0.05 level of significance was used to develop the final multi-variable model. Only those factors which remained associated with MDD at p < 0.05 were retained in the final model.

### Ethical considerations

Approvals were obtained from Makerere University College of Health Sciences Science and Ethical Committee. We also secured ethical approval from the Uganda National Council for Science and Technology. Study approvals from all participating district authorities were also secured. Informed consent was obtained from all study respondents after explaining the objectives and procedures of the study. Informed consent was secured from the respondents before they could be subjected to the study tools. Written consent was secured from those (respondents) who could read and write. For respondents who could not read and write, we used verbal consent. In addition to the verbal consent, they could put their thumb print on the consent paper. Study respondents found to have significant psychiatric problems were referred to the health centre III or IV [16]. The parent interventional study had helped establish a psychiatric service in all health centre III and IV’s in the survey sub-counties. Respondents were assured of confidentiality before the start of each interview. All data collected was kept under key and lock and only accessible to the research team. Personal identifiers were not used at data entry.

## Results

### Study population

Fieldwork was conducted between 2^nd^ January 2013 and 2^nd^ June 2013 where a total of 2,406 respondents were enrolled into this study (819 from Amuru district, 770 from Gulu district and 817 from Nwoya district). At analysis, a total of 45 records were excluded because of missing data on key variables, the excluded records did not differ significantly from those retained on district, age and sex.

### Socio-demographic characteristics of the respondents

Information in this study is adduced from a total sample of 2361 population of 1475 (62.5%) females and 886 (37.5%) males. Table 1, the study participants were distributed in nearly equal proportions among the age groups of 18-24 years (552, 23.8%), 25-34 years (628,27.1%), 35-44 years (479, 20.7%) and those 45 years and above (659, 28.4%). Majority of respondents 658 (69.1%) were married with only 172 (7.2%) single or separated. Majority of respondents 1423 (59.9%) had only attained a primary level education with only 393 (16.5%) having attained a secondary level education and above. On religion, majority were Catholics 1872 (78.2%) with Protestants 304 (12.7%) and Moslems 12 (0.5%). In terms of employment, majority were peasant farmers at 1917 (80%) with only 32 (1.3%) employed as salary earners.

**Table 1:** **Socio-demographic correlates of Major Depressive Disorder in the Wayo-Nero study population, Northern Uganda**

	**Number in study**	**Current major depressive disorder (n)**	**Unadjusted OR (95% CI)**	**Adjusted OR** ^**§**^ **(95% CI)**	**P-value**
	**(N, %)**	**n, (n/N%)**			
**Sex**					
Male	886 (37.5)	151 (17.0)	1	1	
Female	1475 (62.5)	431 (29.2)	2.0 (1.63-2.48)	2.12 (1.71-2.63)	<0.001
**Age (Years)**					
18-24	552 (23.8)	95 (17.2)	1	1	
25-34	628 (27.1)	147 (23.4)	1.47 (1.10-1.96)	1.43 (1.07-1.92)	0.017
35-44	479 (20.7)	125 (26.1)	1.70 (1.26-2.30)	1.71 (1.26-2.33)	0.001
45+	659 (28.4)	215 (32.6)	2.33 (1.76-3.08)	2.33 (1.76-3.09)	<0.001
**Marital Status**					
Currently married/cohabiting	1649 (69.1)	385 (23.4)	1	1	
Widowed	294 (12.3)	112 (38.1)	2.02 (1.55-2.63)	1.55 (1.15-2.09)	0.004
Separated/Divorced	172 (7.2)	64 (37.2)	1.95 (1.40-2.71)	1.95 (1.38-2.74)	<0.001
Single	273 (11.4)	32 (11.7)	0.44 (0.30-0.64)	0.62 (0.39-0.98)	0.039
**Education**					
No education	569 (23.9)	184 (32.3)	1	1	
Primary only	1423 (59.6)	368 (25.9)	0.73 (0.59-0.90)	1.02 (0.80-1.29)	0.874
Secondary/above	393 (16.5)	45 (11.5)	0.27 (0.19-0.39)	0.55 (0.36-0.82)	0.004
**Religion**					
Catholics	1872 (78.2)	472 (25.2)	1	1	
Protestants	304 (12.7)	68 (22.4)	0.85 (0.64-1.14)	0.85 (0.69-1.38)	0.291
Muslims	12 (0.5)	2 (16.7)	0.59 (0.13-2.72)	-	
SDA/Pentecostal/Other	207 (8.6)	56 (27.1)	1.10 (0.80-1.52)	0.98 (0.69-1.38)	0.888
**Employment**					
Peasant farmer/Fisherman	1917 (80.0)	508 (26.5)	1	1	
housewife	129 (5.4)	32 (24.8)	0.92 (0.61-1.38)	0.80 (0.52-1.22)	0.292
Professional/clerical	32 (1.3)	3 (9.4)	0.29 (0.09-0.95)	0.41 (0.12-1.38)	0.151
Tradesperson/artisan/transport	56 (2.3)	11 (19.6)	0.68 (0.35-1.32)	0.96 (0.47-1.96)	0.909
Student/ unemployed but able	137 (5.7)	10 (7.3)	0.22 (0.11-0.42)	0.36 (0.17-0.77)	0.008
Unemployed and disabled	80 (3.3)	22 (27.50)	1.05 (0.64-1.74)	0.76 (0.44-1.30)	0.312
Others	44 (1.8)	9 (20.5)	0.71 (0.34-1.49)	1.09 (0.51-2.34)	0.825

Key: §Adjusted for sex and age.

### Prevalence of major depressive disorder (MDD)

The prevalence of major depressive disorder (MDD) in this study population was 24.7% (95% CI: 22.9%-26.4%). The distribution by gender was 17.0% (95% CI: 14.6%-19.5%) for males and 29.2% (95% CI: 26.9%-31.5%) for females.

### Socio-demographic correlates of MDD

Table 1, shows the socio-demographic correlates for MDD. The following socio-demographic factors were significantly associated with MDD: female gender 2.1(95% CI: 1.71-2.63); increasing age when compared with those in the age group of 18-24 years: 25-34 years age group 1.43 (95% CI: 1.07-1.92), 35-44 years age group 1.71 (95% CI: 1.26-2.33) and 45+ years age group 2.33 (95% CI: 1.76-3.09); being widowed 1.55 (95% CI: 1.15-2.09) and separated/divorced 1.95 (95% CI: 1.38-2.74) when compared with those who were married; and being a student/ ‘unemployed but able bodied’ was protective against MDD 0.36 (95% CI: 0.17-0.77) when compared against being a peasant farmer.

### Social correlates for major depressive disorder (MDD) at multi-variable analysis

Table 2, shows the social factors that were correlated with MDD. The social factors that were significantly associated with MDD were: food insecurity 1.99 (95% CI: 1.61-2.45); self-report of being HIV positive 2.63 (95% CI: 1.87-3.70); low social support 0.41(95% CI: 0.34-0.51), increasing negative life events scores, with those reporting 6-9 events having an aOR of 2.97 (95% CI: 2.19-4.04), those reporting 10-18 events having an aOR of 7.26 (95% CI: 5.17-10.20) when all were compared with those reporting 0-5 events; increasing negative life events stress scores, those with scores of 6-10 had an aOR of 4.99 (95% CI: 2.71-9.19) , those with scores of 11-15 had an aOR of 9.16 (95% CI: 4.97-16.88) and those with scores of 16+ had an aOR of 20.37 (95% CI: 10.95-27.87) when all were compared with those with zero scores; increasing war trauma event scores, those reporting 1-3 events had an aOR of 1.92 (1.34-2.74), those reporting 4-7 events had an aOR of 2.83 (1.97-4.08) and those reporting 8+ events had an aOR of 4.68 (3.26-6.73) when all were compared with those reporting no war trauma event; and increasing war trauma stress scores, with those reporting medium scores having an aOR of 2.07 (95% CI: 1.48-2.89), those reporting high scores an aOR of 4.42 (95% CI: 3.13-6.25) when all were compared with those reporting low scores.

**Table 2:** **Social correlates of Major Depressive Disorder in the Wayo-Nero study population, Northern Uganda**

	**Number in study**	**Major depressive disorder**	**Unadjusted OR**	**Adjusted OR** ^**§**^	**P-value**
	**(N, %)**	**(n, %)**	**(95% CI)**	**(95% CI)**	
**Food Security**					
Enough	1002 (43.3)	173 (17.3)	1	1	
Not enough	1310 (56.7)	407 (31.1)	2.16 (1.76-2.65)	1.99 (1.61-2.45)	<0.001
**Reported HIV Status**					
Negative	1798 (75.9)	403 (22.4)	1	1	
Positive	164 (6.9)	74 (45.1)	2.85 (2.04-3.96)	2.63 (1.87-3.70)	<0.001
Don’t know	407 (17.2)	114 (28.0)	1.35 (1.06-1.72)	1.17 (0.89-1.52)	0.261
**Social support index**					
Low (< median)	1176 (49.0)	398 (33.8)	1	1	
High (Median plus)	1223 (51.0)	200 (16.4)	0.38 (0.31-0.46)	0.41 (0.34-0.51)	<0.001
**Negative life events index**					
Low (0-5 events)	611 (25.4)	66 (10.8)	1	1	
Medium (6-9 events)	1347 (56.0)	334 (24.8)	2.72 (2.05-3.62)	2.97 (2.19-4.04)	<0.001
High (10-18 events)	448 (18.6)	199 (44.4)	6.60 (4.81-9.05)	7.26 (5.17-10.20)	<0.001
**Negative life events stress index**					
0	260 (10.8)	13 (5.0)	1	1	
1-5	517 (21.5)	59 (11.4)	2.45 (1.32-4.55)	2.20 (1.14 -4.22)	0.018
6-10	710 (29.5)	149 (30.0)	5.05 (2.81-9.07)	4.99 (2.71-9.19)	<0.001
11-15	546 (22.7)	188 (34.4)	9.98 (5.56-17.91)	9.16 (4.97-16.88)	<0.001
16+	373 (15.5)	190 (50.9)	19.73 (10.90-35.71)	20.37 (10.95-37.87)	<0.001
**War trauma events index**					
None	410 (17.4)	47 (11.5)	1	1	
1-3 events	763 (32.3)	155 (20.3)	1.97 (1.386-2.798)	1.92 (1.340-2.739)	<0.001
4-7 events	576 (24.4)	167 (29.0)	3.15 (2.215-4.489)	2.83 (1.971-4.078)	<0.001
8+ events	612 (25.9)	213 (34.8)	4.12 (2.916-5.830)	4.68 (3.255-6.727)	<0.001
**War trauma stress index**					
Low	429 (18.2)	49 (11.4)	1	1	
Medium	1128 (47.8)	251 (22.3)	2.22 (1.597-3.084)	2.07 (1.480-2.893)	<0.001
High	804 (34.1)	282 (35.1)	4.19 (3.009-5.833)	4.42 (3.130-6.245)	<0.001

Key: §Adjusted for sex and age.

### Clinical and psychological factors associated with major depressive disorder

Table 3, shows the psychological and clinical factors associated with MDD. The factors that were significantly associated with MDD were: positive coping style (protective against MDD), with those with medium scores having an aOR of 0.23 (95% CI: 0.18-0.30), those with high scores having an aOR of 0.11 (95% CI: 0.08-0.14) all compared with those with low scores; past psychiatric history 3.64 (95% CI: 2.62-5.05); increasing childhood trauma scores, those with a score of 3 having an aOR of 2.07 (95% CI: 1.66-2.59), those with a score of 4-5 having an aOR of 3.27 (95% CI: 2.04-5.23) all compared with those with a score of 1-2; and a family history of mental illness 1.65 (95% CI: 1.33-2.06). Additionally, the following psychiatric disorders/problems were significantly associated with MDD: life-time attempted suicide 13.52 (95% CI: 3.78-48.33); PTSD 4.79 (95% CI: 3.65-6.29); generalized anxiety disorder 7.48 (95% CI: 5.81-9.63) and alcohol dependency disorder 1.51 (1.01-2.26).

**Table 3:** **Psychological and clinical correlates of Major Depressive Disorder in the Wayo- Nero study population, Northern Uganda**

	**Number in study (N,%)**	**Major depressive disorder (n,%)**	**Unadjusted OR (95% CI)**	**Adjusted OR§(95% CI)**	**p-value**
**Positive Coping Style index**					
Low	479 (19.9)	284 (59.3)	1	1	
Medium	705 (29.3)	164 (23.3)	0.21 (0.16-0.27)	0.23 (0.18-0.30)	<0.001
High	1222 (50.8)	151 (12.4)	0.10 (0.08-0.12)	0.11 (0.08-0.14)	<0.001
**Past Psychiatric history**					
No	2156 (90.1)	485 (22.5)	1	1	
Yes	173 (7.2)	89 (51.5)	3.65 (2.66-5.00)	3.64 (2.62-5.05)	<0.001
Don’t Know	64 (2.7)	22 (34.4)	1.80 (1.07-3.05)	1.54 (0.89-2.68)	0.127
**Childhood Trauma index**					
1-2	1668 (70.7)	353 (21.2)	1	1	
3	608 (25.8)	193 (31.7)	1.73 (1.41-2.13)	2.07 (1.66-2.59)	<0.001
4-5	85 (3.6)	36 (42.4)	2.74 (1.75-4.28)	3.27 (2.04-5.23)	<0.001
**Family history of psychiatric illness**					
No	1800 (76.1)	410 (22.8)			
Yes	566 (23.9)	180 (31.8)	1.58 (1.28-1.95)	1.65 (1.33-2.06)	<0.001
**Life-time attempted suicide**					
No	2389 (99.3)	585 (24.5)	1		
Yes	17 (0.7)	14 (82.4)	14.39 (4.12-50.25)	13.52 (3.78-48.33)	<0.001
**Posttraumatic Stress disorder**					
No	2122 (88.2)	447 (21.1)	1		
Yes	284 (11.8)	152 (53.5)	4.31 (3.34-5.57)	4.79 (3.65-6.29)	<0.001
**Generalized anxiety disorder**					
No	2051 (85.2)	372 (18.1)	1		
Yes	355 (14.8)	227 (63.9)	8.00 (6.27-10.22)	7.48 (5.81-9.63)	<0.001
**Alcohol dependency disorder**					
No	2244 (93.7)	554 (24.7)	1		
Yes	162 (6.7)	45 (27.8)	1.17 (0.82-1.68)	1.51 (1.01-2.26)	0.046

### Multivariate analysis of correlates of major depressive disorder

According to Table 4 the following risk factors retained statistical significance with MDD at multivariate analysis: female gender, self-report of being HIV positive, low social support scores, increasing number of negative life events, increasing negative life events stress scores, decreasing positive coping style scores and the psychiatric comorbidities of life-time attempted suicide, PTSD and generalized anxiety disorder.

**Table 4:** **Multivariate model of risk factors for major depressive disorder Disorder in the Wayo-Nero study population, Northern Uganda**

	**Final model**		
	**Odds ratio**	**(95% CI)**	**P-value**
**Sex** (Baseline group – Male)			
Female	1.63	1.25-2.11	<0.001
**Age-Group** (Baseline group – 18-24)			
25-34	1.06	0.75-1.52	0.728
35-44	1.37	0.95-1.98	0.090
45+	1.33	0.94-1.87	0.102
**Self-reported HIV Status**			
Negative/Don’t Know			
Positive	1.83	1.22-2.74	0.003
**Social Support score** High (Median and above)			
(Low - < median)	1.35	1.05-1.75	0.018
**Life events stress index** (Baseline group – 0)			
1-5	1.77	0.83-3.78	0.140
6-10	2.74	1.27-5.89	0.010
11-15	4.26	1.96-9.28	<0.001
16-48	6.63	2.80-15.66	<0.001
**Negative life events** (Baseline group: 0-5 events)			
6-9 events	1.61	1.05-2.44	0.027
10-18 events	1.60	0.91-2.82	0.099
**Positive Coping Style index** (Baseline –Low)			
Medium	0.28	0.21-0.39	<0.001
High	0.19	0.14-0.26	<0.001
**Life-time attempted suicide**			
Present	6.18	1.53-25.01	0.011
**Post-Traumatic Stress Disorder (PTSD)**			
Present	1.90	1.37-2.65	<0.001
**Generalized anxiety disorder (GAD)**			
Present	4.09	3.06-5.49	<0.001

### Association between clinical and behavioural outcomes with major depressive disorder

Table 5, shows the association between MDD and various clinical and behavioural outcomes. Not receiving anti-retroviral therapy for those with HIV was the only outcome that was significantly associated with MDD aOR3.22 (95% CI: 1.08-9.57).

**Table 5:** **Association between Major Depressive Disorder and clinical and behavioral outcomes as seen in the Wayo-Nero study population, Northern Uganda**

	**Number in study (N,%)**	**Major depressive disorder (n,%)**	**Unadjusted OR (95% CI)**	**Adjusted OR§ (95% CI)**	**p-value**
**Whether currently receiving treatment for psychiatric illness** (n = 173)					
No	159 (91.9)	81 (50.9)	1		
Yes	14 (8.1)	8 (57.1)	1.284 (0.426-3.870)	1.103 (0.349-3.480)	0.868
**Whether receiving HIV treatment** (n = 164)					
Yes	139 (84.8)	59 (42.5)	1		
No	25 (15.2)	15 (60.0)	2.034 (0.854-4.845)	3.218 (1.082-9.570)	0.036*
**Whether receiving treatment for the epilepsy** (n = 66)					
No	40 (61.6)	11 (27.5)	1		
Yes	26 (39.4)	9 (34.6)	1.396 (0.481-4.049)	1.789 (0.536-5.973)	0.344
**High risk sexual behaviour** (n = 139)					
No	2223 (94.2)	545 (24.5)	1		
Yes	139 (5.8)	37 (26.8)	1.128 (0.765-1.664)	1.374 (0.905-2.086)	0.136

Key: §Adjusted for sex, age and education.

## Discussion

In this study we investigated the burden of major depressive disorder (MDD) and its association with risk factors and negative outcomes as seen in northern Uganda seven years after the cessation of one of the worst conflicts in the world. The principal finding of this study is that post-conflict northern Uganda still has high rates of MDD although these seem to have reduced in the post-conflict time period. There is still poor uptake of mental health services. This can largely be attributed to the general lack of health services and other factors that affect uptake of mental health services such as traditional beliefs and stigma [24]. The risk factors for MDD fell under the broad domains of socio-demographic factors (female gender, increasing age, being widowed and being separated/divorced); distal psychosocial vulnerability factors (being HIV positive, low social support, increasing war trauma events previously experienced, war trauma stress scores previously experienced, past psychiatric history, family history of mental illness, negative coping style, increasing childhood trauma scores, life-time attempted suicide, PTSD, generalized anxiety disorder and alcohol dependency disorder) and proximal psychosocial stressors (food insufficiency, increasing negative life event scores and increasing stress scores). ‘Not receiving anti-retroviral therapy’ for those who were HIV positive was the only negative clinical and behavioral outcome associated with MDD in the study.

In this study there were more females (62.5%) than males (37.5%) with MDD. Roberts and colleagues [8] in war conflict northern Uganda observed a similar gender ratio. A similar gender ratio is reported by Sheikh et al. in their study among internally displaced persons in Kaduna, north of Western Nigeria [36]. The majority of respondents were less than 45 years of age (71.6%), employed as peasant farmers (80%) and married (69.1%). There was however a considerable proportion who were widowed (12.3%) and separated/divorced (7.2%) all potential consequences of chronic war conflict. In this study 6.9% of the respondents self-reported that they were HIV positive with the majority (84.8%) reporting that they were receiving HIV/AIDS care. The self-reported prevalence of HIV in this study is close to the figure of 8.3% for HIV sero-prevalence for the mid- northern Uganda region (area where the survey was conducted) [37]. About a tenth (7.3%) of the respondents self- reported that they had ever suffered from a nervous or psychiatric problem of these only 8% reported that they had ever sought treatment for this condition from a health facility indicating that many of such cases are not reported to the modern health system. Kinyanda and colleagues in non-war affected south-western Uganda reported a prevalence of self-reported severe mental illness of 0.9% [27]. Other Ugandan studies [38,39] have noted that less than 20% of all persons with mental illness are treated at formal health services in the country. Closing the treatment gap for mental illness (as indicated in this study) is a challenge in poor communities such as northern Uganda [40].

The prevalence of major depressive disorder (MDD) in this study was 24.7%. This prevalence figure did not change much (increased to 29.1%) when a similar criteria used in previous studies in northern Uganda was applied to the data (where MDD was defined as meeting only the symptom criteria of the MDD module in the MINI excluding the function assessment dimension). In previous studies undertaken during the conflict in northern Uganda using non diagnostic assessment tools, prevalence figures for MDD of between 45% and 67% were reported which are about twice those observed in this study [5,8]. This difference in the figures reported during the conflict and seven years after the conflict (period for the current study) suggest that the rates of MDD in northern Uganda may have reduced after the conflict. We obtained low prevalence rates largely due to two factors; a) we undertook MDD assessment using a diagnostic criteria and therefore we were more likely to get low prevalence rates. Kuwert et al. [41] urges that approaches that use symptom assessment normally provides presumptive rates of prevalence of mental health conditions [41] b) prevalence rates normally change over time.

On risk factors of MDD in this study, these will be discussed under socio-demographic factors, social factors and psychological and clinical factors. Most of the socio-demographic factors significantly associated with MDD in this study were indices of socio-economic disadvantage in this post-conflict situation in northern Uganda, these were: female gender, increasing age, being widowed, separated and divorced, and having no formal education, with female gender retaining statistical significance in the final multivariate analysis. Previous studies in conflict and post-conflict settings have reported the following socio-demographic factors to be associated with MDD: female gender [2-4,8,12,14]; increasing age [42]; being widowed, separated and divorced [2,8,43] and having no formal education [44].

The following social factors were significantly associated with MDD in this study: food insufficiency, being HIV positive, poor social support, increasing number of negative life events, increasing life events stress scores, increasing number of reported war trauma events and increasing war trauma events stress scores. As communities are working towards recovery, they still grapple with the long term effects of the war on the individual, family and the entire community system. In the final multivariate model HIV positive status, poor social support, increasing number of negative life events and the associated stress scores retained statistical significance. Previous studies in conflict settings have reported the following social factors as significantly associated with MDD: degree of exposure to traumatic events [2-5,8,13,15,16]; negative life events [13]; and poor social support [45,46]. From studies undertaken in non-conflict settings HIV positive status [47] and food insufficiency [44] have been reported to be significantly associated with MDD.

The following psychological and clinical factors were significantly associated with MDD in this study: negative coping style, past psychiatric history, childhood trauma, family history of mental illness and the comorbid psychiatric problems of lifetime attempted suicide, PTSD, generalized anxiety disorder and marginally alcohol dependency disorder. At multivariate analysis the psychological and clinical factors that remained significantly associated with MDD were negative coping style and the comorbid psychiatric disorders of PTSD and generalized anxiety disorder. Previous studies in conflict settings have reported negative coping style [48] and psychiatric comorbidities [23,49,50] as risk factors for MMD. Sheikh et al. while focusing on a post-conflict setting in Kaduna Northwestern Nigeria reported the co-morbidity of PTSD and depression (those that had PTSD were more likely to have depression) [51].

Among the investigated negative outcomes, it was only poor uptake of antiretroviral therapy (ART) that was significantly associated with MDD. Gourlay and colleagues [50] in a systematic review observed that MDD was one of the barriers against the uptake of antiretroviral drugs in prevention of mother-to-child transmission of HIV programs in sub-Saharan Africa [51]. Similarly, Olisah et al. [52] reported that 42. 3% of their respondents with depressive disorder did not use their medications regularly [52].

### Study limitations

In this study, MDD was associated with poor uptake of antiretroviral therapy (ART). But given the cross sectional nature of this study, it was not possible to elucidate the direction of causality between these investigated factors and MDD. This could be better understood if this was a longitudinal study.

We were not able to develop a sampling frame because there were no updated lists of community members at community level in most villages. This implies that the total sample might be a true representation of the target population. However using the health centers as a central place, we could fun out in one direction until all households in that direction a completed. We could then turn to another direction and the same process was repeated throughout the study. This meant that households near and far from the roads were included into the sample.

We used Psychiatric nurses to administer the study tools. It is plausible that more accurate data on diagnosis assessment could have been obtained if we had used highly trained cadres in mental health. Though this was unavoidable given the fact that there are few mental health workers in the region of higher qualifications (by the time this study was conducted northern Uganda had only three psychiatrists and the out of three, two had retired and were only working with the University in Gulu). The region also had one clinical psychologist). We however, used a support team of psychologists and psychiatrists from Makerere University to supervise data collection and offer regular training from time to time.

## Conclusion

This study showed that post-conflict northern Uganda still had high rates of MDD. The risk factors for MDD fell under the broad domains of socio-demographic factors (female gender, increasing age, being widowed and being separated/divorced); distal psychosocial vulnerability factors (being HIV positive, low social support, increasing war trauma events previously experienced, war trauma stress scores previously experienced, past psychiatric history, family history of mental illness, negative coping style, increasing childhood trauma scores, life-time attempted suicide, PTSD, generalized anxiety disorder and alcohol dependency disorder) and proximal psychosocial stressors (food insufficiency, increasing negative life event scores, increasing stress scores) in line with the stress-vulnerability model of depression [36].

Overall, given the high level of vulnerability in the study areas to MDD due to a number of factors (including psychiatric, psychological and social factors) there is the need for effective multi-sectoral programs tailored towards addressing the high rates of MDD in the region. Longitudinal studies are recommended to continuously assess the trend/rates of MDD in the region and remedial action taken.
